# Global Longitudinal Strain Alteration of the Left Ventricle in Children with Organic Aciduria: Cardiac Disease in Organic Aciduria

**DOI:** 10.3390/jcm15041393

**Published:** 2026-02-10

**Authors:** Bastien Moysset, Célia Hoebeke, Brigitte Chabrol, Guillaume Carles, Beatrice Desnous, Julie Delphine Blanc, Caroline Ovaert, Fedoua El Louali

**Affiliations:** 1 Pediatric and Congenital Cardiology Department, AP-HM Timone Enfants, 13385 Marseille, France; 2 Center for Inherited Metabolic Diseases, Filière G2M, La Timone University Hospital, 13385 Marseille, France; 3 Marseille Medical Genetics, Inserm UMR 1251, Aix Marseille University, 13331 Marseille, France; 4 Laboratory of Biomechanics and Application, UMRT24, Gustave Eiffel University, Aix Marseille University, 13005 Marseille, France

**Keywords:** organic aciduria, propionic acidemia, methylmalonic academia, cardiomyopathy, echocardiography, cardiac strain

## Abstract

**Introduction:** Cardiac complications are well-documented in propionic acidemia (PA), and there are a few reported cases of cardiomyopathies in methylmalonic acidemia (MMA). Left-ventricular global longitudinal strain (LV GLS) measurement is known to be able to detect early ventricular dysfunction, leading potentially to cardiomyopathy. The aim of our study was to evaluate left-ventricular global longitudinal strain (LV GLS) in MMA and PA patients and compare it with the pediatric general population. **Methods:** In this monocentric retrospective study, 26 patients with organic aciduria (OA) were included. Demographic, clinical, electrocardiographic and echocardiographic data were collected. The mean LV GLS in MMA and PA patients was compared with the GLS in the pediatric general population. **Results:** The left-ventricular ejection fraction (LVEF) was similar between MMA and PA patients and in the normal range (66.27 ± 6.24% vs. 61.41 ± 11.02%; *p* = 0.182). LV GLS was significantly lower in PA patients than in MMA patients (−15.8 ± 5.67% vs. −20.6 ± 3.19%; *p* = 0.011). LV GLS was significantly lower in PA patients when compared with the general pediatric population (*p* = 0.029). **Conclusions:** Patients with propionic acidemia may have impaired global longitudinal strain even in the presence of normal LVEF. LV GLS might be a useful tool for cardiac follow-up in pediatric patients with OA.

## 1. Introduction

Organic acidurias (OAs), more commonly named organic acidemias, form a heterogeneous group but are well-defined hereditary metabolic pathologies. The main mechanism in OA is deficient mitochondrial breakdown of coenzyme A-activated carbonic acids, causing mitochondrial dysfunction with subsequent energy impairment, impaired mitophagy and altered post-translational protein acylation [1,2], ultimately leading to acute and/or chronic dysfunction of organs with a high energy demand [2,3]. Propionic acidemia (PA) and methylmalonic acidemia (MMA) are the most common organic acidurias [1].

### 1.1. Pathophysiology of Organic Acidemia

PA and MMA are autosomal recessive diseases characterized by a rare disorder of propionate catabolism, caused respectively by an enzymatic deficiency in propionyl-CoA carboxylase and methyl malonyl-CoA mutase [4].

Certain forms of MMA are due to a deficiency of cobalamin (i.e., vitamin B12) [4].

MMA has an estimated incidence of ~ 1: 50,000 and PA of ~ 1:100,000–150,000 [4,5].

Organic propionic and methylmalonic acidurias are characterized by the accumulation in the body of propionate derivatives.

Propionate is synthesized by the body via three main sources: firstly, degradation of certain amino acids such as valine, isoleucine, threonine and methionine; secondly, fermentation of the bacterial flora of the digestive tract called “propiogenic”; and finally, lipolysis via certain long-chain fatty acids [1,4].

Both PA and MMA lead to the accumulation of metabolites in the urine, respectively, 3-hydroxypropionic acid and methyl citric acid in PA, and methylmalonic acid in MMA. Those organic acids can be measured in the urine by chromatography, hence the name of organic aciduria. This accumulation leads to an array of endogenous toxicity exerted on many organs (the brain, liver, kidneys, heart, skin, pancreas, and hematopoietic system) and also leads to a profound and ubiquitous dysfunction of mitochondrial metabolism.

The progression of these diseases includes acute metabolic decompensation, but also progressive organ dysfunction. In PA, a progressive disease may be observed, including cognitive impairment and movement disorders, myopathy, pancreatitis, and cardiac pathologies, including cardiomyopathy (CM) and acquired long QT syndrome (aLQTS) [4]. Furthermore, acute cardiac events (arrhythmic and myocardial) can occur during metabolic crises. Cardiac disease significantly contributes to morbidity in individuals with PA.

While cardiac complications (cardiomyopathies and acquired long QT syndrome) are well-known in PA, cardiomyopathies are less frequently reported in MMA [6,7,8]. Both dilated and hypertrophic cardiomyopathy have been reported in both diseases [6,7,8,9,10,11]. Regular echocardiographic and electrocardiographic surveillance is, hence, recommended for all patients with propionic and methyl malonic aciduria [4].

Regarding therapeutic management of OA, except for vitamin B12-responsive forms of MMA, the outcome remains poor despite apparently effective therapy, including a low-protein diet and supplements of L-carnitine and ammonium. From a cardiological perspective, prevention of cardiac events does not appear to be completely ensured by conventional therapies [12]. Numerous scientific reports highlight the interest of liver transplantation in reversing CM in PA [13,14,15,16]. The high mortality level of liver transplant [17] and the possibility of CM recurrence [18] following such a challenging intervention are also discussed.

### 1.2. Echocardiographic Assessment of the Left Ventricle (LV) in Organic Aciduria

Conventional echocardiography assessing systolic LV function by measurement of LV fractional shortening (LV FS) is the most frequently used technique for cardiac systolic dysfunction evaluation in PA [9,12,13].

Currently, more sensitive echocardiographic techniques have been developed, allowing for a reliable assessment of diastolic function (using tissue Doppler imaging) and systolic function (via measurement of LV global longitudinal strain (LV GLS) quantified by speckle-tracking echocardiography (STE)). These techniques have improved cardiac function evaluation in several genetic diseases, such as Duchenne or Becker muscular dystrophy [19,20] and acquired pathologies following treatment of childhood cancer [21].

*1. Structure and Histology of the Left Ventricle*

The left ventricle has a well-studied but, nevertheless, complex histological structure [22,23]. Longitudinal muscle fibers form the inner (endocardial) and outer (epicardial) layers, while circumferential fibers lie between the two previous ones [24]. LV contraction results from the combined action, including circumferential and longitudinal shortening, radial thickening, and inverse rotational movement between the apex and base during systole [23].

*2. Left-ventricular ejection fraction (LVEF)*

Left-ventricular (LV) function, assessed by cardiac ultrasound, is a major prognostic factor of heart disease. Indeed, LVEF remains a milestone in therapeutic decisions related to myocardial performance. Thus, various LVEF thresholds are pertinent to the initiation of cardioprotective pharmacotherapies and device therapies in several cardiac diseases [25,26]. The most commonly used method for determining LV function by cardiac ultrasound is left ventricular ejection fraction (LVEF), calculated as the ratio of ejection LV volume divided by the LV end-diastolic volume. However, this method only reflects the circumferential and radial function of the LV and can be inaccurate or imprecise [27,28]. For example, in concentric left-ventricular (LV) hypertrophy with a small LV chamber size, LVEF can be normal despite left-ventricular dysfunction since normal EF may hide a small stroke volume. Moreover, the most commonly used imaging modality to assess LVEF is two-dimensional echocardiography. This modality has inherent limitations relating to LV geometric assumptions and LV cavity border tracing [29].

Nevertheless, LVEF still remains a correct and largely used risk predictor. In fact, reductions in LVEF portend worse cardiovascular outcomes [30]. This relationship is conserved until a plateau at an EF of 40% to 45%, above which LVEF is unrelated to mortality [31]. All these elements demonstrate the limitations of the LVEF.

*3. Two-dimensional strain*

Several ultrasound techniques analyzing myocardial deformation have been developed over the last 30 years. Among them, the two-dimensional strain method, 2D-strain or “two-dimensional speckle-tracking echocardiography” (2-DSTE), has emerged [27,28,32]. This technique is now available on most ultrasound machines intended for routine echocardiography. The 2D strain corresponds to the change in length of myocardial fibers between end-diastole and end-systole and is expressed as a percentage [33]. Its measurement requires simultaneous recording of the heart rate. The ultrasound image is analyzed in segments; each group of pixels is called a “speckle” and corresponds to an acoustic marker.

The movement of this “speckle” is followed in several directions during the cardiac systole, with subsequent quantification of the movement relative to the initial position [33]. Quantification of the myocardial deformation allows the analysis of the myocardial contraction at the segmental and global level and on the different axes: longitudinal, radial and circumferential [33,34].

Among all 2D strain measurements, LV global longitudinal strain (LV GLS) is one of the most robust, validated and reproducible parameters [28,35]. It is obtained by calculating the average of the measurements obtained from three apical sections of the LV. A normal LV GLS has a negative value because it corresponds to shortening of the longitudinal fibers during systole. The more negative the number is, the better the heart muscle function.

LV GLS and its normal values are now well defined and validated in the adult and general pediatric populations [36,37,38], and it is considered to be more sensitive than the measurement of LVEF to assess LV function [4,39].

LVEF analysis takes little account of the longitudinal component of left-ventricular systolic function. In many heart diseases, the first function to deteriorate is this longitudinal deformation, which is easily evaluated by the 2D strain [28]. As a result, patients may, at an early stage, have a normal LVEF but alteration of myocardial 2D strain [39].

There is a lack of data regarding prognosis in children, but the adult literature is abundant concerning risk prediction using global longitudinal strain. GLS is correlated to mortality, independent of and incremental to LVEF in adult heart failure [40]. Stanton et al. confirmed in a meta-analysis that GLS is a stronger predictor than LVEF of all-cause mortality and a composite of cardiac death, HF hospitalization, and malignant arrhythmias [41].

### 1.3. The Aim of This Study

The aim of this study was to extensively evaluate LV function in MMA and PA patients using all standard measurements, including the 2-DSTE method, and to compare the measurements in the two populations and with available normal pediatric data.

## 2. Method

### 2.1. Study Population

This monocentric retrospective study was conducted at La Timone Children’s University Hospital, Marseille, France. All patients with an OA diagnosis (MMA and PA) were enrolled.

The La Timone Children’s Hospital Clinical Investigation Committee and the ethics committee approved the study protocol. All methods were performed in accordance with the relevant guidelines and regulations and with the Declaration of Helsinki.

The exclusion criteria were clinically unstable patients at the time of the echocardiography; a poor ultrasound window; and patients with arrhythmia at the time of the echocardiography. Twenty-six consecutive pediatric patients with OA were included.

### 2.2. Data Collection

Data collection was completed between October 2024 and January 2025. Demographic characteristics (age, gender, and weight), clinical data (number of metabolic decompensations, history of congestive heart failure, history of fatigue, retrosternal chest pain upon exertion and presence of an asymptomatic murmur, and arrhythmias), electrocardiographic (ECG) abnormalities, and transthoracic echocardiography (TTE) findings focusing on left-ventricular function were collected.

### 2.3. Echocardiographic Data Collection

Transthoracic echocardiography exams were routinely performed by senior echocardiologists (specialized in pediatric cardiology) using a PHILIPS EPIQ CVx echocardiography machine (Koninklijke Philips, The Netherlands). Echocardiographic records were reviewed and interpreted simultaneously by two echocardiographers (pediatric cardiologists). The intra-observer variability and agreement of LV strain parameters were assessed using the interclass correlation coefficient (ICC), and the strength of agreement was based on the ICC value. The ICC was estimated as excellent (ICC = 0.91) for observer 1 and good (ICC = 0.89) for observer 2.

***Left-ventricle diastolic function*** was assessed by the following.

The early to late diastolic transmitral flow velocity (E/A) ratio (obtained in a 4-chamber view with pulsed wave Doppler), early diastolic mitral annular tissue velocity (e′) (obtained in a 4-chamber view with tissue Doppler imaging), E/e’ ratio and indexed left-atrial area (iLAA). The inferior vena cava diameter was used to determine patient volume status.

***Left-ventricular systolic function*** was assessed using the following:

- The M-mode (collecting diastolic interventricular septal thickness (IVS), diastolic posterior wall thickness (PWT), end-diastolic diameter (EDD), and end-systolic diameter (ESD)).
- The Simpson biplane left-ventricular ejection fraction (LV EF), where volume measurements were based on tracings of the blood–tissue interface in the apical four- and two-chamber views. The LV end diastolic volume (LV EDD) was assessed with the Simpson method, indexed to body surface area.
- Visual assessment of global and regional myocardial function, where each segment was analyzed individually in multiple views.
- LV global longitudinal strain (LV GLS) was measured and calculated from segmental averaging of the three apical views: the apical 4-, 3-, and 2-chamber views.

Off-line analysis was performed using the QLAB version 10.5 application (Philips Healthcare, Andover, MA, USA) by the two sonographers. The data were reviewed, analyzed and collected retrospectively by an independent physician. Age-referenced ranges of LV GLS were defined as established by Lévy et al. [42]. Values below the 95% confidence interval were considered abnormal. GLS values below −18% were considered as dysfunctional since it is a clinically accepted cut-off for subclinical dysfunction [43].

### 2.4. Statistical Analysis

Statistical analysis was performed using the SPSS software, version 17.0 (SPSS, Inc., Chicago, IL, USA).

Quantitative variables were expressed as means ± standard deviations (SDs) for normal distribution variables and as medians and interquartile ranges [IQRs] for non-normal distribution variables. Qualitative variables were expressed as numbers and percentages. For normally distributed variables, comparison of qualitative data between groups was performed by the chi-square test (or Fisher’s exact test in case of sizes < 5), and comparison of continuous data was performed by using Student’s *t*-test. For non-normally distributed variables, comparison of qualitative data between groups was performed by the nonparametric chi-square test (or Fisher’s exact test). The comparison of continuous data was made using the Mann–Whitney test.

All clinical measurements, such as height, weight, and body mass index, were converted to a Z-score using the growth chart [44]. We converted all diameters and lengths of cardiac structures to a Z-score using the Z-score of Cardiac Structures of Detroit Data [45]. The mean LV GLS values in our patients were compared with reported reference values by Lévy T. et al. [42] in the general pediatric population, using the Levene test.

All *p*-values were bilateral, and significance was pronounced for a *p*-value of less than 5%. Statistical analysis was performed using the SPSS software, version 22.0 (SPSS, Inc., Chicago, IL, USA).

## 3. Results

### 3.1. Demographic and Clinical Characteristics

A total of twenty-six patients were screened for this study. Eleven patients had an MMA diagnosis, and fifteen had a PA diagnosis. One MMA patient died during the follow-up.

The diagnosis of AO was made during the first month of life in 15 patients (57.7%). For patients without neonatal diagnosis, the median age at diagnosis was 8.5 [4–15.7] months. The demographic and clinical characteristics are presented in Table 1. Gender, age, weight Z-score, height Z-score, body mass index Z-score, systolic, diastolic and mean blood pressure were statistically similar between the PA and MMA populations. There was no difference between the two groups with regard to the number of metabolic decompensations. All patients had dietary treatment, amino acid supplementation, and L-carnitine supplementation. In the MMA group, seven patients (63.6%) had vitamin B12-responsive forms of MMA. There were more liver and kidney transplants in the MMA group compared with the PA group (*p* = 0.039).

At cardiac evaluation, all patients were metabolically stable with at least three months’ delay between the last decompensation and the echocardiogram. Patients with kidney disease were examined in the year following a kidney transplant (four patients) or at the end hemodialysis session (seven patients).

Three patients had enalapril treatment for cardiac dysfunction. Of those patients, one was symptomatic with stage II dyspnea. All other patients were asymptomatic.

### 3.2. Electrocardiographic Data

Table 2 shows the electrocardiogram parameters in both populations. No electrocardiogram anomalies were noted, such as prolonged QTc interval or arrhythmias (supraventricular or ventricular tachycardia or ventricular fibrillation). Sixteen patients underwent Holter monitoring. There were no major rhythmic events. Three patients experienced minor events: two atrial extrasystoles and one ventricular extrasystole.

### 3.3. Echocardiographic Data

All echocardiograms were obtained in sinus rhythm. The quality of echocardiographic records was good and adequate for analysis in all patients. Only 21 segments of a total of 442 segments were excluded due to a tracking issue.

- *Comparison between the two groups (PA* vs. *MMA)*

The LVEF was similar in both groups (PA vs. MMA) and within normal limits. Three patients (20%) in the PA group had impaired LVEF, while 10 (66.7%) had abnormal LV GLS. In the MMA group, no patient had impaired LVEF, while three (27.3%) had abnormal LV GLS.

GLS was significantly lower in PA patients than in MMA patients (−15.8 ± 5.67% vs. −20.6 ± 3.19% respectively, *p* = 0.011). All three components of GLS, S4C, S2C and S3C were lower in PA patients than in the MMA population. The results are presented in Table 3.

- *Comparison with the general pediatric population*

**Figure 1:**
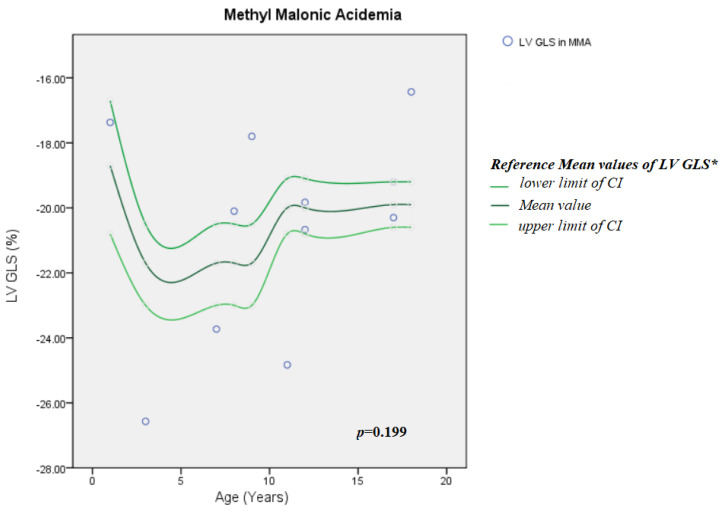
LV GLS in MMA group compared with general pediatric population according to age. CI = confidence interval; LV GLS = left-ventricular global longitudinal strain; MMA = methyl malonic acidemia; *: reference values, as reported by Levy et al. [42].

**Figure 2:**
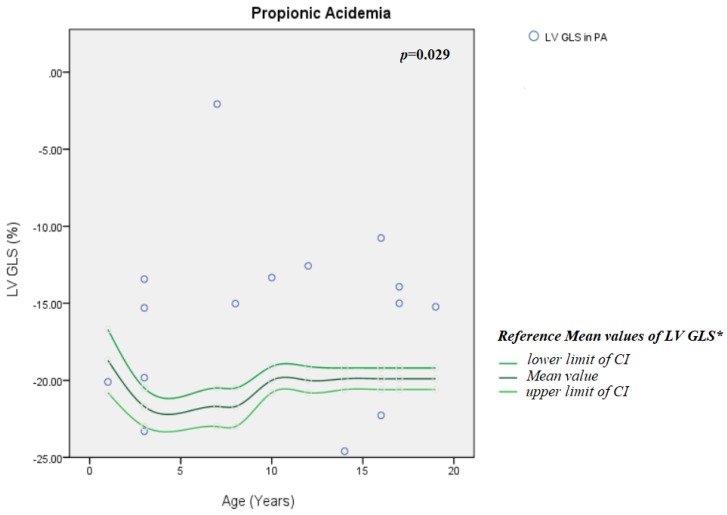
LV GLS in PA group compared with general pediatric population according to age. CI = confidence interval; LV GLS = left-ventricular global longitudinal strain; PA = propionic acidemia. *: references values, as reported by Levy et al. [42].

There was no difference in GLS between MMA patients and the general pediatric population (*p* = 0.199) (Figure 1). GLS in PA patients was significantly lower compared with the general population (*p* = 0.029) (Figure 2).

- *Comparison between altered and normal LV GLS patients*

There was no statistically significant difference between the altered LV GLS group and the normal LV GLS group regarding the number of metabolic decompensations (2.5 [1–6] vs. 4 [1.75–7.5]; *p* = 0.41) nor the age of the patient (10.2 ± 6.35 vs. 8.7 ± 5.12; *p* = 0.54). PA was associated with more altered LV GLS compared with MMA (66.7% vs. 27.3%; *p* = 0.047).

## 4. Discussion

According to our findings, GLS was significantly lower than normal in the PA group, indicating impaired LV systolic function in PA patients, while LVEF, the most used cardiac function index, remained normal in both the MMA and PA populations. These findings are consistent with the data found in the literature.

### 4.1. LV GLS in Organic Acidemia

In a cohort of 18 patients, Kovacevic A. et al. [46] found that PA patients had a decrease in LV GLS, as in our population. However, the vast majority of these patients also had an abnormal LVEF and already had symptoms of heart failure, which was not the case in our patients. Our patients may be at an earlier stage of cardiac dysfunction, showing the potential interest of the method for detecting early cardiac lesions.

Liu Y. et al. found an alteration in LV GLS in MMA patients compared with healthy subjects (22.7 ± 1.8 for the control group vs. 20.3 ± 2.6 for isolated MMA and 21.3 ± 1.9 for MMA with homo-cystinuria; *p* < 0.001) [47]. We did not observe such an alteration in our population with MMA. The reason can be twofold: firstly, the limited number of patients studied (n = 11) did not make it possible to highlight a difference, even a minimal one. But also, we compared our population with the lower limits of known reference values, while Liu et al. compared their population with controls of the same age and detected a difference that was certainly significant, but with values that could remain within the normal range [47].

### 4.2. Cardiomyopathy in Organic Aciduria

Cardiomyopathy has been observed more commonly in patients affected by PA [48].

The exact physiopathology of cardiomyopathy in propionic acidemia and methylmalonic acidemia still remains unknown. Moreover, as described in our study, cardiac lesions can develop regardless of the severity of metabolic derangement [13]. Scientific data suggest that heart lesions are due to an accumulation of toxic metabolites [49,50], leading to mitochondrial dysfunction [51] with impaired mitochondrial energy metabolism, increasing levels of cellular oxidative stress [52,53,54], and gene expression changes [12,13,55,56,57,58]. Indeed, several miRNAs have been found to be elevated in mice and patients with PA. These miRNAs are known to play critical roles in heart health and disease [55,56,57,58,59].

Histological studies of cardiac tissue (post-heart transplant or post-mortem) revealed coenzyme Q10 deficiency in Baruteau et al.’s case report [59] and low tissue free carnitine and NADH cytochrome c reductase in Mardach et al.’s case report [11]. Despite these reports, clinical studies have not consistently demonstrated the efficacy of coenzyme Q10 [12] or carnitine supplements’ [10] beneficial effects for cardiac disease related to PA.

The diagnosis of cardiomyopathy associated with propionic acidemia is seen in up to 23% of the patients in series, with a median age of discovery varying from 7 to 14.4 years [12,13], indicating an onset of the disease in childhood and adolescence. Early detection of cardiac dysfunction can lead to treatment decisions and may change the prognosis of this disease [21].

Transthoracic cardiac ultrasound is performed regularly and easily to monitor patients with organic aciduria. “Two-dimensional speckle tracking” or 2-DSTE offers precise evaluation of myocardial deformation and can identify preclinical cardiac dysfunction, even when LVEF is within normal values [39].

A decrease in LV GLS between two examinations in the same patient and in the same load conditions must be interpreted as a true alteration of myocardial function [28] and be considered as a detection of myocardial damage at an early stage. This hypothesis requires longitudinal follow-up and dedicated prospective studies to assess the accuracy of GLS as a risk marker of cardiomyopathy in OA. Further studies may allow us to develop medical strategies for cardiac prevention.

### 4.3. Limitations of the Study

Our study, however, had certain limitations.

The size of our sample population was an important limitation. A larger sample size could allow us to detect even more subtle echocardiographic abnormalities in patients with organic aciduria. The limited number of patients studied also restricted the performance of in-depth subgroup analyses. Under this condition, the risk of type I error is real, forbidding us from over-interpreting nominally significant results. Thus, confidence intervals seem to be more informative than *p*-values alone.

Our study also suffered from the retrospective and monocentric nature of the collection of data, leading to potential selection bias.

Our patient cohort exhibited marked demographic and clinical heterogeneity. Therefore, we sought to express and convert most of our data into standardized values and Z-scores and to compare LV GLS values with those of the general pediatric population by age group [42] in order to minimize the impact of confounding factors. Our study included patients from a single center, without a healthy control group. Although comparison with reference GLS values from the literature is acceptable, these cohorts often differ in terms of acquisition protocol and software.

Longer-term follow-up of those echocardiographic parameters will likely be of great interest to better understand the evolution of the cardiac impairment. Indeed, a prospective follow-up assessing the accuracy of GLS alterations to predict subsequent clinical cardiomyopathy is required. Secondly, the evaluation of potential beneficial effects of therapeutic agents such as beta-blockers, antagonists of the renin–angiotensin–aldosterone system or gliflozins could be performed. Finally, it would have been relevant to simultaneously study other cardiac evaluation parameters, such as NT pro BNP testing, exercise testing or other imaging modalities, such as myocardial scintigraphy or cardiac. Further studies regarding this topic are needed.

## 5. Conclusions

Our findings suggest that patients with propionic acidemia have impaired LV GLS even before LVEF becomes abnormal, indicating early LV systolic dysfunction. Adding systematic use of LV GLS measurement to standard cardiological monitoring could benefit patients with organic aciduria and allow earlier detection of the onset of cardiac subclinical anomalies. Further prospective studies could assess the accuracy of this parameter in early detection of cardiac dysfunction. Should the accuracy of this parameter in early detection of cardiac dysfunction be confirmed, cardiological therapies such as neurohormonal blockers (angiotensin-converting enzyme inhibitors, aldosterone agonist beta-blockers, etc.) may be proposed during these early and subclinical stages of cardiac dysfunction, as proposed in other conditions (such as in cancer therapy-related cardiovascular toxicity [21]). Further studies to evaluate the effect of these therapies on LV GLS alteration will be interesting.

## Figures and Tables

**Table 1:** Baseline characteristics between MMA and PA groups at echocardiographic exam.

Characteristics	MMA Group n = 11	PA Group n = 15	*p*
Age (years), mean ± SD	10.4 ± 5.6	9.9 ± 6.3	0.830
Male gender, n (%)	5 (45.5)	8 (53.5)	0.18
Weight Z-score, mean ± SD	−1.4 ± 1.2	−0.7 ± 0.9	0.115
Height Z-score, mean ± SD	−2.1 ± 1.2	−1.6 ± 0.6	0.240
Body surface area (m^2^), mean ± SD	0.90 ± 0.28	0.96 ± 0.4	0.699
SBP (mmHg), mean ± SD	114.0 ± 8.1	104.1 ± 12.5	0.054
DBP (mmHg), mean ± SD	68.5 ± 7.8	63.9 ± 10.1	0.257
HR (bpm), mean ± SD	102.4 ± 23.7	101.1 ± 26.6	0.899
Number of metabolic decompensations, n [IQR]	3 [1.5–6]	3 [1–6]	0.838
Liver and/or Kidney transplantation, n (%)	6 (54.5)	1 (6.67)	0.039
Hemoglobin (g/dL), mean ± SD	11.6 ± 1.23	12.4 ± 1.29	0.159
Interval echocardiogram/last decompensation (months), median [IQR]	18 [9.75–24]	12 [5–36]	0.858

BMI: body mass index; SBP: systolic blood pressure; DBP: diastolic blood pressure; MBP: mean blood pressure; HR: heart rate; IQR: interquartile range; SD: standard deviation.

**Table 2:** Electrocardiogram parameters of MMA and PA groups.

Parameters	MMA Group n = 11	PA Group n = 15	*p*
P-R interval (ms), mean ± SD	125.0 ± 18.8	137.2 ± 30.1	0.24
QRS interval (ms), mean ± SD	80.4 ± 9.8	88.1 ± 16.3	0.17
QTc interval (ms), mean ± SD	411.0 ± 35.8	404.9 ± 34.7	0.70
Extrasystoles, n (%)	0 (0)	3 (2)	0.225

SD: standard deviation.

**Table 3:** Comparison of echocardiographic data.

Echocardiographic Data	MMA Group n = 11	PA Group n = 15	*p*
EF (%) mean ± SD	66.27 ± 6.24	61.41 ± 11.02	0.182
EF < 50%, n (%)	0 (0)	3(20.0)	0.001
iLV EDV	69.8 ± 17.7	68.7 ± 20.8	0.906
S4C (%), mean ± SD	−21.2 ± 3.89	−14.4 ± 7.11	0.005
S2C (%), mean ± SD	−21.1 ± 3.89	−16.8 ± 6.31	0.047
S3C (%), mean ± SD	−20.4 ± 3.60	−16.2 ± 6.32	0.049
GLS (%), mean ± SD	−20.6 ± 3.19	−15.8 ± 5.67	0.011
GLS < −18%, n (%)	3 (27.3)	10 (66.7)	0.047
Abnormal LV GLS, n (%)	3 (27.3)	10 (66.7)	0.047
LVIDD Z-score, median [IQR]	−0.41 [−0.77–1.02]	0.62 [−0.25–1.1]	0.341
IVS Z-score, median [IQR]	0.61 [0.21–0.96]	−0.32 [−0.75–0.55]	0.020
LVPWD Z-score, median [IQR]	0.1 [−0.14–1.87]	0.41 [−0.47–0.67]	0.393
E/A ratio, mean ± SD	1.61 ± 0.39	1.71 ± 0.37	0.582
E/E’ ratio, mean ± SD	6.73 ± 1.22	6.42 ± 0.79	0.527
iLAA (cm^2^/m^2^), mean ± SD	8.92 ± 1.89	9.31 ± 2.12	0.673
IVC diameter (mm), mean ± SD	7.55 ±2.51	8.31 ± 3.68	0.585

LV: left ventricle; RV: right ventricle; EF: ejection fraction; iLV EDV: indexed left ventricle end diastolic volume; S4C: strain in apical 4-chamber apical view; S2C: strain in apical 2-chamber view; S3C: strain in apical 3-chamber apical view GLS: global longitudinal strain; LVIDD: left-ventricular internal diastolic diameter; IVS: interventricular diastolic septum; LVPWD: left-ventricular posterior wall diastolic length; FS: fractional shortening; E/A: ratio of early and late diastolic mitral inflow velocity; E/E’: ratio of early diastolic mitral inflow velocity to early diastolic mitral annulus velocity; iLAA: indexed left-atrial area; IVC: inferior vena cava; IQR: interquartile range; SD: standard deviation.

## Data Availability

Data available on request due to restrictions (ethical reasons).
